# First demonstration of the circulation of a pneumovirus in French pigs by detection of anti-swine orthopneumovirus nucleoprotein antibodies

**DOI:** 10.1186/s13567-018-0615-x

**Published:** 2018-12-05

**Authors:** Charles-Adrien Richard, Caroline Hervet, Déborah Ménard, Irina Gutsche, Valérie Normand, Fanny Renois, François Meurens, Jean-François Eléouët

**Affiliations:** 1 Unité de Virologie et Immunologie Moléculaires, INRA, Université Paris-Saclay, 78350 Jouy-en-Josas, France; 2 BIOEPAR, INRA, Oniris, Université Bretagne Loire, 44307 Nantes, France; 3 CNRS, CEA, IBS, University of Grenoble Alpes, 38000 Grenoble, France; 4 Porc.Spective Swine Vet Practice, Chêne Vert Conseil Veterinary Group, ZA de Gohélève, 56920 Noyal-Pontivy, France

## Abstract

The presence of pneumoviruses in pigs is poorly documented. In this study, we used the published sequence of the nucleoprotein (N) of the recently identified Swine *Orthopneumovirus* (SOV) to express and purify SOV N as a recombinant protein in *Escherichia coli*. This protein was purified as nanorings and used to set up an enzyme-linked immunosorbent assay, which was used to analyse the presence of anti-pneumovirus N antibodies in swine sera. Sera collected from different pig farms in the West of France and from specific pathogen free piglets before colostrum uptake showed indirectly that a pneumovirus is circulating in pig populations with some variations between animals. Piglets before colostrum uptake were sero-negative for anti-pneumovirus antibodies while most of the other pigs showed positivity. Interestingly, in two farms presenting respiratory clinical signs and negative or under control for some common respiratory pathogens, pigs were detected positive for anti-pneumovirus antibodies. Globally, anti-pneumovirus N antibody concentrations were variable between and within farms. Further studies will aim to isolate the circulating virus and determine its potential pathogenicity. SOV could potentially become a new member of the porcine respiratory complex, important on its own or in association with other viral and bacterial micro-organisms.

**Electronic supplementary material:**

The online version of this article (10.1186/s13567-018-0615-x) contains supplementary material, which is available to authorized users.

## Introduction

Recently, the former paramyxoviral subfamily *Pneumovirinae* was elevated to family status *Pneumoviridae* [1–3]. This new family is composed of the two genus *Metapneumovirus* and *Orthopneumovirus*. Seven different viruses have been identified in several animal species as belonging to this family: avian metapneumovirus (AMPV) and human metapneumovirus (HMPV) for the *Metapneumovirus* genus, bovine respiratory syncytial virus (BRSV), ovine respiratory syncytial virus (ORV), human respiratory syncytial virus (HRSV), pneumonia virus of mice (PVM), and canine pneumovirus (CPV) for the *Orthopneumovirus* genus. More recently, an eighth pneumovirus was identified by metagenomic sequencing of pooled nasal swabs in feral swine in the USA [4]. This newly identified *Orthopneumovirus* shows 93% and 91% protein identities with PVM and CPV, respectively, and was named swine *Orthopneumovirus* (SOV). Since no specific enzyme-linked immunosorbent assay (ELISA) is available for SOV, based on the close genetic relationship between PVM and SOV Hause et al. used a commercial ELISA to detect antibodies to PVM and found that 31% of the analysed feral swine sera were antibody positive [4]. Finally, analyses by the same PVM ELISA of sera from different American farms revealed that sera were 33% to 93% positive, dependent on the farms, and confirmed that SOV is also present in domestic swine. Interestingly, using bovine respiratory syncytial virus antigens, in 1998 Allan et al. found that 41% of pigs sera from 61 herds in Northern Ireland were reactive with BRSV antigens [5].

Although SOV has not yet been isolated, the complete genomic sequence of SOV (strain 57) is available (GenBank accession number: KX364383.1). We thus used the published sequences to synthesize expression vectors for nucleoprotein (N) and phosphoprotein (P) in order to express these proteins in bacteria. As developed previously with HRSV [6], co-expression of the C-terminal region of SOV P fused to GST together with SOV N allowed us to purify SOV N nanorings. These N nanorings were used further to develop an ELISA in order to detect the presence of anti-pneumovirus N antibodies in domestic pigs in the West of France.

## Materials and methods

### Expression and purification of recombinant SOV nucleoprotein

**Figure 1:**
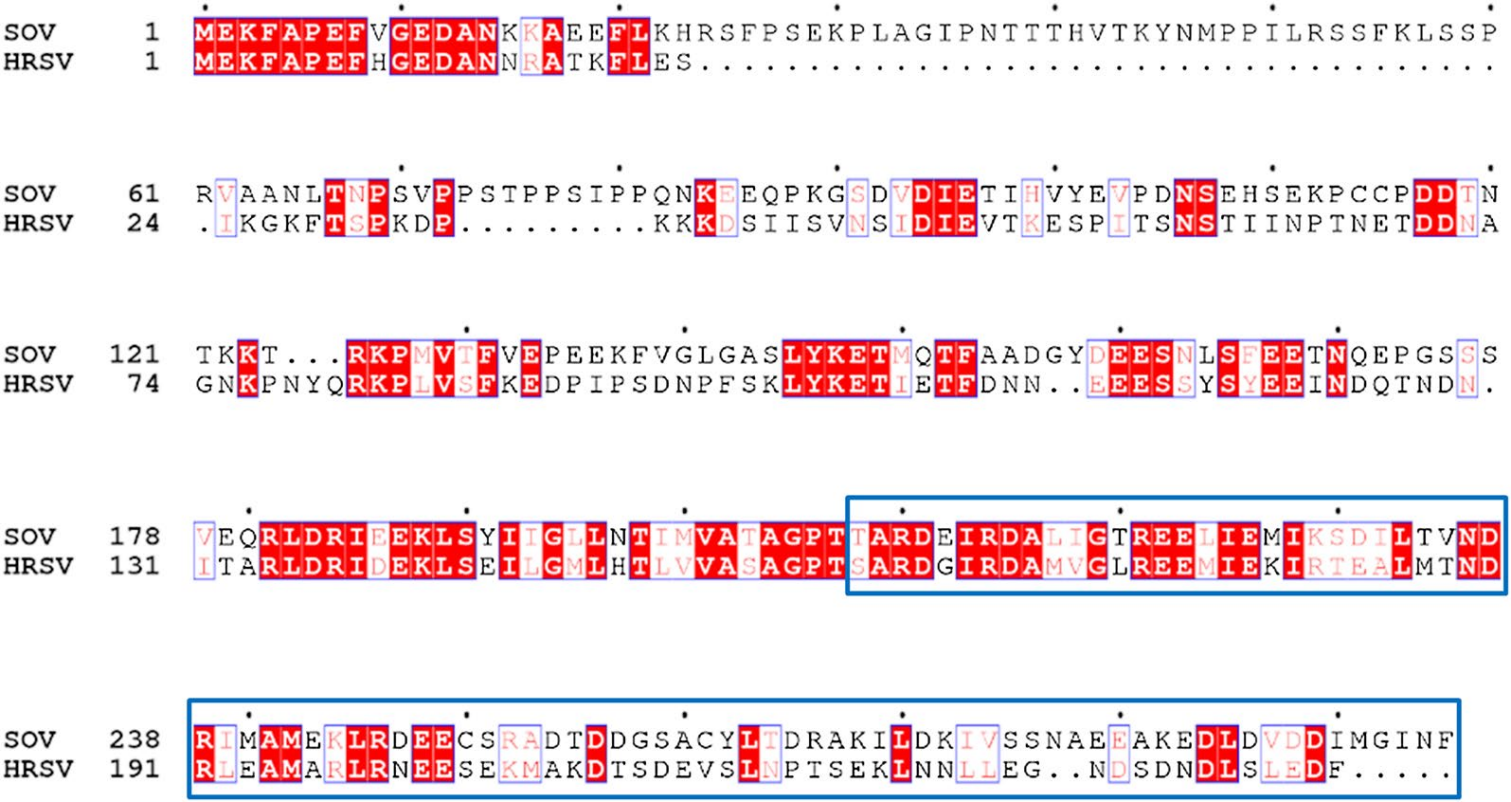
**Amino acid sequence alignments between full-length SOV (strain 57) and human RSV (HRSV, strain Long VR-26) P proteins.** Invariant residues are highlighted in white font on a red background. The C-terminal region of HRSV P identified previously as an N-RNA binding region (residues 161–241) and the corresponding region for SOV P (residues 208–295) are boxed in blue. Genbank accession codes: ANO40516.1 (SOV P) and AHC94770.1 (HRSV). Sequences were aligned with Clustal W and treated with ESPript 3.

The full-length SOV N and P coding sequences (GenBank accession number: KX364383.1) were synthesized for optimized expression in *Escherichia coli* (Genscript). Our previous studies with respiratory syncytial virus showed that the P C-terminal disordered region (PCT, amino acid residues 161–241, Figure 1) fused to GST is very efficient for purifying HRSV N protein after co-expression in *E. coli* [6]. The same approach was applied to SOV. First, the C-terminal disordered region of SOV P (amino acid residues 208–295) was determined by alignment with HRSV P (Figure 1). The identified region of SOV P was amplified by PCR and subcloned in pGEX-4T-3 at BamHI-XhoI sites to engineer the pGEX-PCT vector. SOV N gene was subcloned in pET28a+ at NdeI-XhoI sites to engineer the pET-N vector. *E. coli* BL21 (DE3) (Novagen) cells were co-transformed with the pGEX-PCT and pET-N plasmids and were grown at 37 °C for 8 h in 1 L of Luria–Bertani (LB) medium containing 100 µg/mL ampicillin and 50 µg/mL kanamycin. The same volume of fresh LB was then added, and protein expression was induced by adding isopropyl-ß-d-thio-galactoside (IPTG) to the medium (final concentration 0.33 mM). Bacteria were grown at 28 °C and harvested by centrifugation 15 h after induction. Bacterial pellets were re-suspended in lysis buffer (50 mM Tris–HCl pH 7.8, 60 mM NaCl, 1 mM EDTA, 2 mM DTT, 0.2% Triton X-100, 1 mg/L lysozyme) supplemented with complete protease inhibitor cocktail (Roche, Mannheim, Germany) and incubated for 1 h on ice, sonicated, and centrifuged at 4 °C for 30 min at 10 000 × *g*. Glutathione-Sepharose 4B beads (GE Healthcare, Uppsala, Sweden) were added to clarified supernatants and incubated at 4 °C for 15 h. Beads were then washed two times in lysis buffer and three times in PBS 1X, then stored at 4 °C in an equal volume of PBS. To isolate PCT-N complexes, beads were resuspended in an equal volume of PBS + 1 mM DTT and incubated with biotinylated thrombin (Novagen) for 16 h at 20 °C. Thrombin was then removed using the Thrombin cleavage capture kit according to the manufacturer’s instructions (Novagen). Purified complexes were run in polyacrylamide gels stained with Coomassie blue.

### Negative stain electron microscopy observations of recombinant SOV nucleoprotein

Three microliter of sample were applied to the clean side of carbon on a carbon–mica interface and stained with 2% sodium silicotungstate. Micrographs were recorded on a FEI Tecnai T12 microscope operated at 120 kV with a Gatan Orius 1000 camera. Images were recorded at a nominal magnification of 23 000 × resulting in a pixel size of 2.8 Å.

### Serum samples

*Positive standards*: Sera obtained from different pig farms located in the West of France were used to search for positive reactions to SOV nucleoprotein. Sera with an optical density (OD) superior to 3.5 were selected as positive standards. Amongst these sera, one with intermediate OD was used to make serial dilutions (1/50, 1/75, 1/100, 1/200, 1/400, 1/800, 1/600, 1/3200, and 1/6400) and generate a standard curve. Then, two additional sera with extremely high OD were also assessed to generate potential standard curves.

#### Negative standards

Sera from newborn specific pathogen free piglets (SPF), just before colostrum uptake, generously provided by Anses Ploufragan (France), were used as negative controls (Table 1). SPF sows from Anses are originally delivered by cesarean section in germ free conditions. Then, sows and their piglets are routinely tested by ELISA (serology) and PCR assays against various pathogens (porcine circoviruses, influenza viruses, Aujeszky disease virus, *Mycoplasma hyopneumoniae*, *Haemophilus parasuis*, *Actinobacillus pleuropneumoniae*, porcine reproductive and respiratory syndrome virus, *Pasteurella multocida*, *Bordetella bronchiseptica*, *Streptococcus suis* type 1 and 2, *Salmonella* spp. and others) to confirm their SPF status at Anses high containment pig research facilities.

**Table 1:** **Information about the different pig farms assessed in the study (− negative, + positive)**

Farms	Anses-SPF piglets^a^	A	B	B	C	C	D	E
Age of selected pigs (days)	0	150	84	105	91	105	160	150
Number of animals	5	10	15	12	10	8	15	8
Respiratory symptoms identified in the farm	No	Yes	Yes	Yes	±	±	No	Yes
Respiratory symptoms observed on the batch (Post-weaning or fattening period)								
Cough	No	Yes	Yes	Yes	Yes	Yes	No	Yes
Dyspnoea	No	Yes	Yes	Yes	No	No	No	Yes
Sneezing	No	Yes	Yes	Yes	Yes	Yes	No	Yes
PRRSV in the farm	No	No	No	No	No	No	No	Yes
Mycoplasma hyopneumoniae in the farm	No	Vaccinated	Vaccinated	Vaccinated	Vaccinated	Vaccinated	No	Vaccinated
Influenza virus isolation in post-weaning	No	H1N2 April 2015			ND	ND	No	ND
Influenza status of the batch	–	+	+	+	ND	ND	–	ND
Actinobacillus pleuropneumoniae	–	No specific lesions	No specific lesions	No specific lesions	No specific lesions	No specific lesions	Free for all serotpyes	No specific lesions
SOV serology	–	+	+	+	+	+	+	+

ND: not documented.

^a^Newborn piglets before colostrum uptake.

All the other tested sera were obtained during routine prophylactic monitoring from five different pig farms (A–E) all located in the West of France (Table 1). In three farms (A, B, and E) clinical signs such as coughing, sneezing, and high respiratory frequency were clearly identified in most of the pigs. In one farm (D), faint clinical signs such as coughing were reported in some pigs.

To optimize the reading of every OD, sera were diluted 1/800 as validated from the dilution range analysis (Additional file 1). ELISA to detect the presence of antibodies porcine reproductive and respiratory syndrome virus (PRRSV) (IDEXX Montpellier SAS, Montpellier, France) and *Mycoplasma hyopneumoniae* (IDEXX and Oxoid-ThermoFisher Scientific), two major porcine respiratory pathogens [7], had been performed previously to assess the status of the selected pig farms. Porcine influenza is endemic in most of the French pig farms [8, 9] and the virus was either isolated, not sought or absent. Regarding *Actinobacillus pleuropneumoniae*, specific lesions were sought and in one farm serology to all commonly circulating serotypes assessed. All the pigs were routinely followed by veterinarians for clinical signs of respiratory infection (coughing and increased respiratory frequency amongst others). To test cross-reactivity with BRSV, porcine sera were assessed with IDEXX RSV IgG Ab Test, a commercial ELISA developed to detect IgG targeting BRSV in bovine sera. The six porcine sera with the highest OD in our ELISA and the seven sera with lowest OD were used for the bovine ELISA. As positive and negative controls, bovine sera positive and negative for anti-RSV IgG, generously provided by large animal clinic (Dr Sebastien Assié, Oniris), were used. Then, the same bovine samples were tested in our next described anti-SOV ELISA.

### Enzyme-linked immunosorbent assays

Swine orthopneumovirus specific antibodies were detected using an ELISA with SOV N as target antigen. Optimal concentrations of the antigen and the enzyme conjugated secondary antibody were defined after testing different conditions as specified in the recommendations for standardization and validation of ELISA assay [2]. ELISA plates (NUNC 96, ThermoFisher Scientific, Illkirch, France) were coated overnight at 4 °C with SOV N (15 µg/mL) in carbonate buffer (0.1 M, pH 9.5, 100 µL/well). The plates were then washed twice for 5 min with phosphate-buffered saline (PBS) containing 1% Tween 20 (Sigma-Aldrich Chimie, Saint-Quentin Fallavier) (PBST) and blocked by an incubation with 130 µL/well of 2% gelatin (Bio-Rad, Marnes-la-Coquette, France) in carbonate buffer (0.1 M, pH 9.5) for 30 min at 37 °C. After this incubation, the liquid was discarded and plates were washed twice with PBST. Then, 100 µL of each serum sample, serially diluted in PBST to dilution 1/800, was added (in duplicate) and incubated at 37 °C for 1 h. Subsequently, the plates were washed twice with PBST and incubated for 1 h at 37 °C with 100 µL/well of goat anti pig IgG conjugated to horseradish peroxidase (Goat anti Pig IgG (Fc): HRP ref AAI41P) (Bio-Rad) at 0.1 µg/mL diluted in PBST. Then, the plate was washed twice with PBST and twice with PBS. Substrate solution, TMB signal + (Bio-Rad) was subsequently added (100 µL/well) and the plate was incubated at 22–27 °C for 20 min. The reaction was stopped by the addition of 100 µL H_2_SO_4_ 0.2 M to each well (Sigma-Aldrich Chimie). Optical densities (OD) were measured at 450 nm using a microplate reader (ThermoFisher Scientific Multiskan GO 1.01.10).

## Results

### Purification and characterization of recombinant SOV N protein

**Figure 2:**
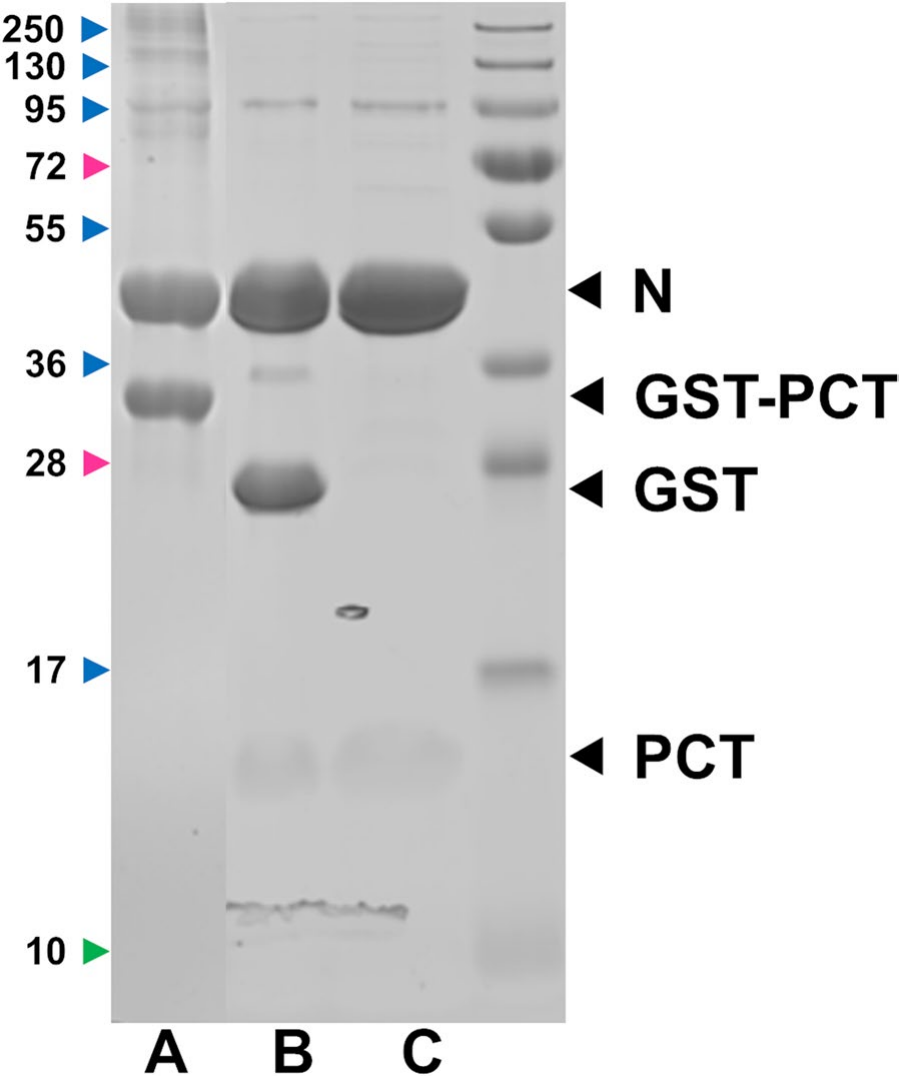
**SDS-PAGE analysis of GST-PCT–N complexes purified from** ***E. coli***. Samples were denatured in Laemmli buffer, run on 10% polyacrylamide gels and detected with Coomassie brilliant blue staining. The PCT–N complexes bound to glutathione-Sepharose beads were incubated with thrombin overnight at 20 °C. After centrifugation, beads or supernatant were loaded (2 µL per lane). A = beads before cleavage; B = beads after cleavage; C = Supernatant containing soluble PCT + N; protein molecular size standards (kDa) are on the left.

We previously developed a protocol to purify HRSV N protein as nanorings containing 10 or 11 N protomers and 70 or 77 bases in length RNA, respectively [6, 10]. HRSV P is composed of a N-terminal disordered region (residues 1–125), a central tetramerization domain (residues 126–160) and a C-terminal disordered region (residues 161–241) [11]. The C-terminal region of HRSV P (PCT) fused to GST was found highly efficient for purifying RSV N from *E. coli*. In order to apply this technique to SOV, we aligned HRSV and SOV P protein sequences to define the corresponding regions of SOV P. Thanks to sequence homologies between HRSV and SOV P, the SOV P region extending from amino acid residues 208 to 295 (MW ~10 kDa) was identified as the C-terminal disordered and N-binding region of P (Figure 1). This SOV PCT region was fused to GST in the pGEX-4T-3 vector and co-expressed in *E. coli* together with the pET-28-N vector. As shown in Figure 2, SOV N and GST-PCT were purified to > 95% homogeneity. SOV N, migrating with apparent MW of 43 kDa, as expected, was recovered as a soluble protein after thrombin cleavage of the GST-PCT/N complexes bound to glutathione-Sepharose beads.

**Figure 3:**
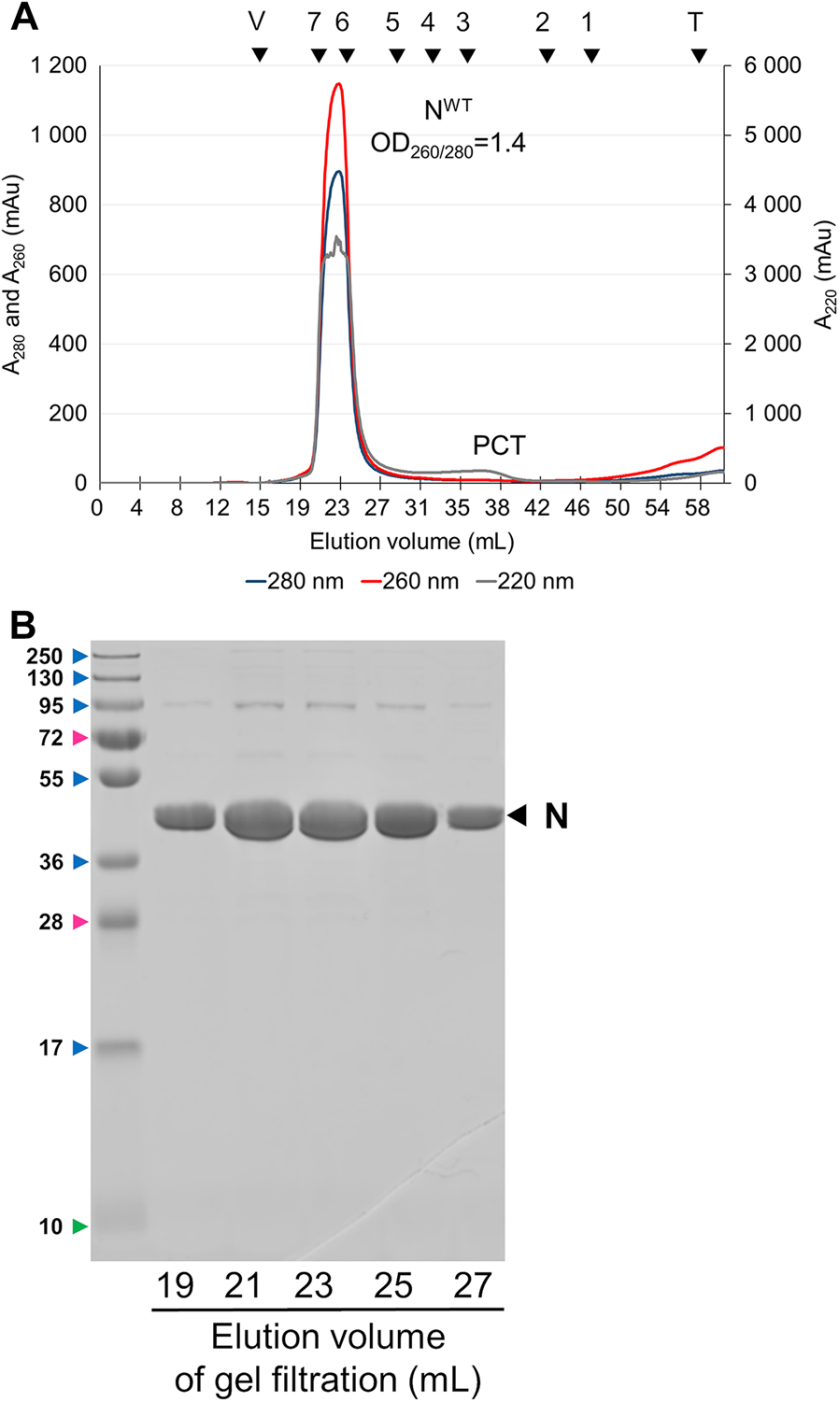
**Elution profile of the PCTD + N complexes. A** Absorbances were monitored at 280 nm (blue line), 260 nm (red line) and 220 nm (gray line). Samples (3 mL) were filtered through a 0.22 µm pore-size filter and run on a Sephacryl S200 column and fractions of 1 mL were collected. The following size markers (GE Healthcare) were used to calibrate the column (mass in parentheses): 1, ribonuclease A (13 700); 2, chymotrypsinogen A (20 200); 3, ovalbumin (47 200); 4, albumin (61 000); 5, catalase (232 000); 6, ferretin (440 000); 7, thyroglobulin (699 000). V, Void volume; T, total volume of the column. **B** The indicated fractions were analyzed by SDS-PAGE as described in Figure 2.

When using a similar technique, HRSV N protein is recovered as rings containing either 10 or 11 N protomers and with a diameter of ~15–20 nm [6, 10]. To determine whether SOV N was also composed of nanorings, purified complexes were analysed by size-exclusion chromatography on a Sephacryl S-200 column (GE Healthcare) and the collected fractions were analysed by SDS-PAGE and Coomassie blue staining. The first peak (P1) eluted at 23 mL, with absorption detected at both 220, 260 and 280 nm, and contained exclusively the SOV N protein (Figure 3). Since the UV absorption was higher at 260 nm than 280 nm, these data indicated that RNA was present in the N fraction. The size of this complex was estimated to be approximately 530 kDa, indicating that N was present as oligomers. The second peak (P2) only absorbed at 220 nm and contained the PCT fragment. Its apparent mass was comprised between 25 and 43 kDa, higher than its predicted mass of 10 kDa, presumably due to a markedly non-globular shape of the P fragment.

**Figure 4:**
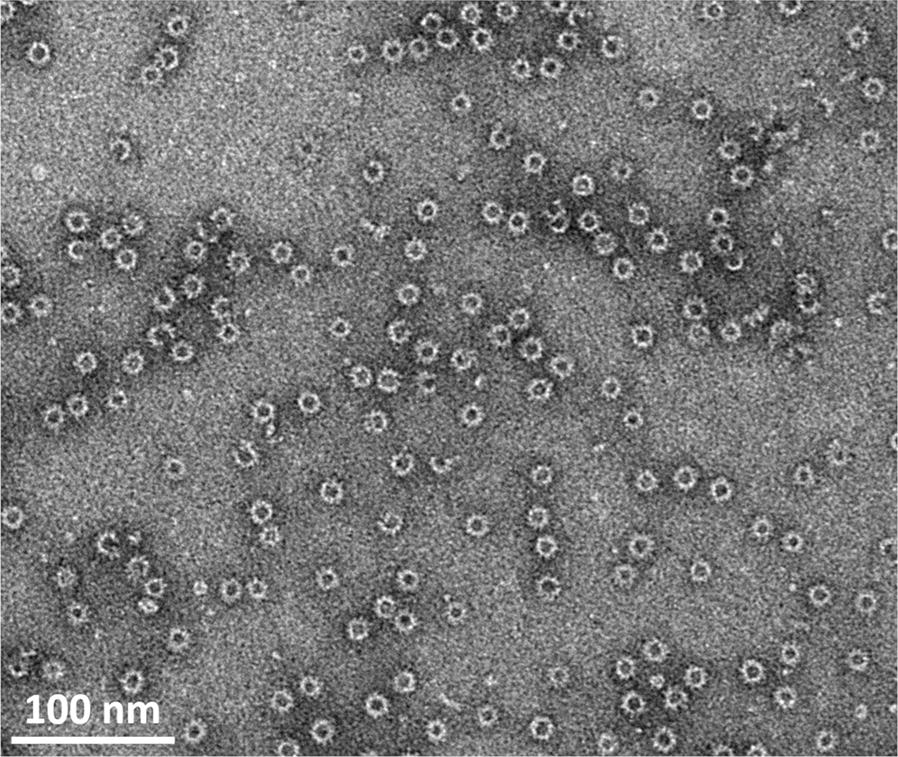
**A typical negative stain electron microscopy image of recombinant SOV N after purification by gel filtration.** Scale bar, 100 nm.

The SOV N protein purified by gel filtration was analysed by negative stain electron microscopy. A representative micrograph, shown in Figure 4, confirmed that SOV N was recovered as nanorings with a diameter of ~15 nm.

### Detection of anti-pneumovirus antibodies in French swine herds using SOV N protein

The recombinant SOV N was then used to develop an ELISA in order to detect antibodies targeting pneumovirus N in some French pig farms. ELISA was optimized and the 1/800 dilution, still in the linear phase of the standard curve (Additional file 1) as recommended [2], was ultimately selected. Indeed, the 1/400 dilution generated too many samples with very OD and was not further used (Additional file 2). Additional standard curves generated from subsequent samples with higher OD needed extremely high dilutions to linearize the signal and were not selected for following analyses (Additional file 3).

**Figure 5:**
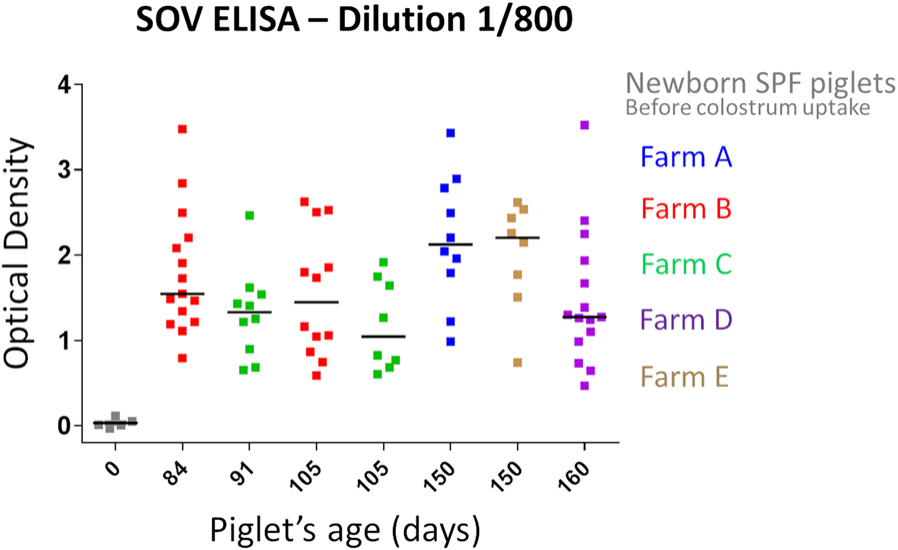
**SOV N ELISA results.** Optical density (OD) was varying between herds with some specific pathogen free piglets before colostrum uptake staying free of anti-pneumovirus antibodies. Dilution 1/800 was selected for the analysis. The characteristics of the selected pig farms are presented in Table 1.

Specific pathogen free piglets before colostrum uptake were sero-negative for anti-pneumovirus N antibodies. On the contrary, most of the sera collected from pigs raised in five different farms from the West of France that were routinely monitored for respiratory infectious diseases (see Table 1) showed positivity to various levels (Figure 5). Variations between pigs positive for anti-pneumovirus N antibodies was significant with OD ranging between 0.47 and 3.52 (dilution 1/800). Three sera presented very high OD values and ideally should have been further diluted, at least two folds, before being tested. Interestingly, a herd (C) negative for PRRSV, without *A. pleuropneumoniae* specific lesions, and presenting mild respiratory symptoms was positive for anti-pneumovirus N antibodies (Figure 5 and Table 1). However, influenza virus status was not determined for this farm (Figure 5).

**Figure 6:**
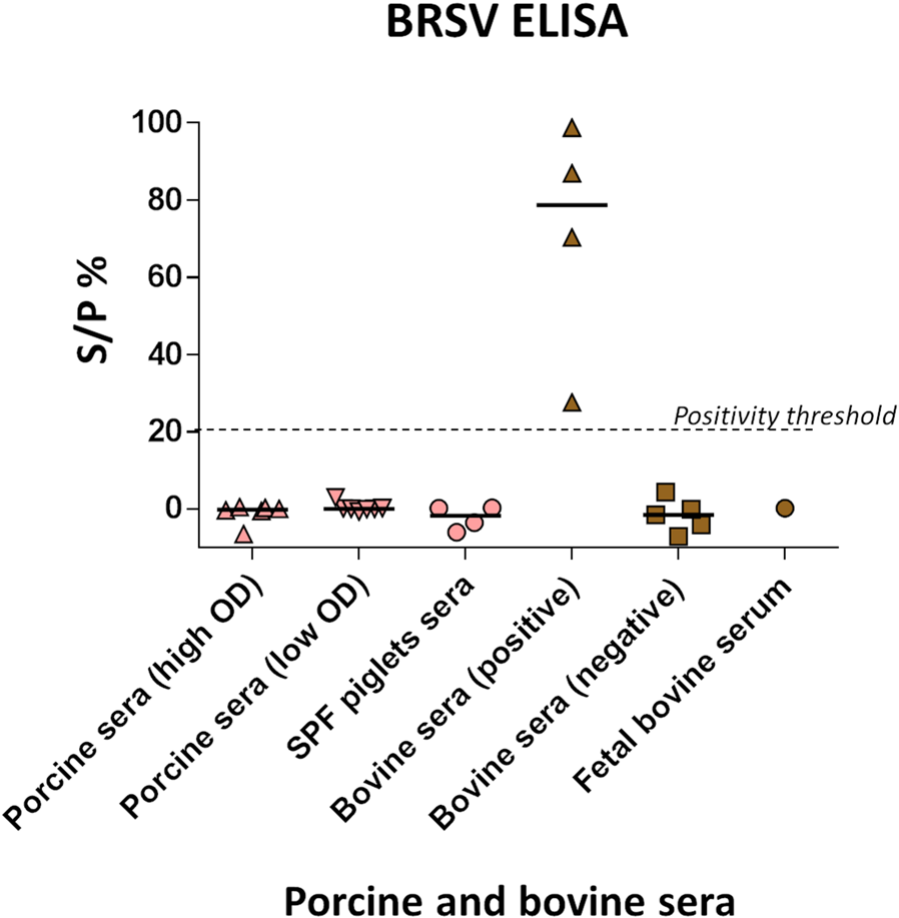
**Commercial BRSV ELISA results.** Selected porcine and bovine sera were tested for the presence of anti-BRSV antibodies. S/P% = 100 × (NE/NExpc). NE: Net Extinction value for each sample was calculated by subtracting the OD450 value of the control well (S–Ag A450) from the OD450 value of the coated well (S + Ag A450) as recommended by the provider.

Cross reactivity tests based on porcine sera tested with the BRSV ELISA and bovine sera tested with our pig ELISA showed absence of cross-reactivity of the antibodies. Indeed, porcine sera tested with the BRSV ELISA could be considered negative for anti-BRSV antibodies (see Figure 6) and strongly positive bovine sera for BRSV had OD values < 0.005 when tested with our new ELISA.

## Discussion

The presence of pneumoviruses in swine is poorly documented. Recently a newly discovered swine pneumovirus (SOV) was identified by metagenomic sequencing of pooled nasal swabs in feral swine in USA [4]. Sequence comparison with other members of the *Pneumoviridae* family showed that SOV is closely related to PVM and CPV. For example, amino acid sequence identities between SOV and other pneumoviruses N proteins are of 97.5% for PVM, 97.5% for CPV, 59.8% for HRSV, 60% for BRSV, 45.7% for HMPV, and 43.3% for AMPV, respectively. However, SOV has not been isolated yet and it is completely unknown whether this virus is pathogenic.

Although a study performed by Allan et al. in 1998 showed that 41% of pigs sera from 61 herds in Northern Ireland were reactive with BRSV antigens [5], thus suggesting the presence of pneumovirus in swine, this kind of study has not been carried out in other European countries yet. The aim of this work was to investigate the presence of pneumovirus in French swine. Among pneumovirus proteins, the N protein is highly conserved between strains and, as an example, for HRSV it was shown to be highly immunogenic either by natural infection [12] or by administration [13–18]. RSV N protein has also been used to develop RSV specific ELISAs [12–18]. Using the recently published sequence of SOV, we engineered and purified recombinant and soluble SOV N protein from *E. coli*. Like observed previously with HRSV [6], SOV N protein produced in bacteria forms soluble nanorings containing nucleic acids that can be purified to > 95% homogeneity by using the C-terminal region of SOV P fused to GST. Like for HRSV [10], the SOV nanorings were composed of two major populations with an average diameter of ~15 nm, which correspond to the diameter of the left-handed helical nucleocapsid of pneumoviruses [10, 19, 20]. Although we have no explanation for the presence of nanorings only and the absence of helical structures in this system, these nanorings must be formed thanks to the elasticity of N oligomers, the absence of other factors such as full length P, and N–N interactions occurring after one helix turn and closing the ring structure.

Swine orthopneumovirus N nanorings were then used to develop an anti-pneumovirus N ELISA. Our results revealed indirectly a significant circulation of a pneumovirus between pigs within selected pig farms in the West of France and an absence of anti-pneumovirus antibodies in SPF piglets. Results in these SPF piglets could be used to estimate the cut-off value for negativity around OD 0.5 even if it must be further confirmed by future research with experimental inoculation of the virus to fully naïve pigs. Moreover, seronegativity of SPF piglets is also an interesting observation as it shows an absence of cross-reactivity of our ELISA test to porcine plasmatic proteins. Then, the absence of cross-reactivity with BRSV allowed us to exclude the potential circulation of a bovine pneumovirus in pigs. However cross-reactivity with other pneumoviruses should also be tested. Indeed, pneumovirus N proteins present strong similarities between pneumoviruses. Our cross-reactivity test results could seem contradictory with the study from Allan et al. [5]. This discrepancy might be explained by the use of various BRSV ELISA (differences in the conformation of the selected antigen and sensitivity for instance). The detection of anti-pneumovirus N antibodies in one farm (C) negative for PRRSV, under vaccination control for *M. hyopneumoniae*, and most probably negative for *A. pleuropneumoniae* but still presenting respiratory symptoms might suggest a potential pathogenicity of SOV in pigs, in association or not with other member of the porcine respiratory complex (PRC). The situation in farms A and B, showing similar respiratory pathogen profile to that of farm C but where influenza virus had been detected before might also support to some extent that hypothesis. However, since many other pathogens are involved in the PRC and because of the absence of clinical data collected for every individual pig in our study, there is an urgent need for further research before being able to formulate strong conclusions regarding SOV potential pathogenicity.

In summary, this work is the first one showing that a pneumovirus is present or had circulated in some French pig farms. Future prospects will be needed to give a more complete image of the percentage of positive pigs in French farms. However, it is currently completely unknown whether SOV is pathogenic or not for pigs. Future isolation of SOV and experimental animal infections are needed to allow deep characterization of the porcine pneumovirus and the determination of its pathogenicity in various conditions. SOV could also represent a new animal model for pneumovirus infection [21].

## Additional files

**Additional file 1.** **Serum dilution range of the SOV N ELISA.** Serum dilution 1/800 were ultimately selected for antibody detection.

**Additional file 2.** **SOV N ELISA results using dilution 1/400.** Optical density (OD) was varying between herds with some specific pathogen free piglets before colostrum uptake staying free of anti-pneumovirus antibodies.

**Additional file 3.** **Standard curves generated from highest OD samples (green and brown lines) were compared to the selected standard curve (blue line).** Dilutions of the negative standard is presented in red.

## Abbreviations

AMPV: avian metapneumovirus
CPV: canine pneumovirus
ELISA: enzyme linked immuno sorbent assay
GST: glutathione-*S*-transferase
HMPV: human metapneumovirus
PVM: pneumonia virus of mice
MW: molecular weight
N: nucleoprotein
OD: optical density
P: phosphoprotein
PCT: phosphoprotein C-terminal region
PRRSV: porcine reproductive and respiratory syndrome virus
RSV: respiratory syncytial virus
SOV: swine orthopneumovirus
TBE: Tris–borate–EDTA buffer
