# Supplementary figures and images for: First demonstration of the circulation of a pneumovirus in French pigs by detection of anti-swine orthopneumovirus nucleoprotein antibodies
Source: Vet Res. 2018 Dec 5;49:118. doi: 10.1186/s13567-018-0615-x (PMC6280484; doi:10.1186/s13567-018-0615-x)

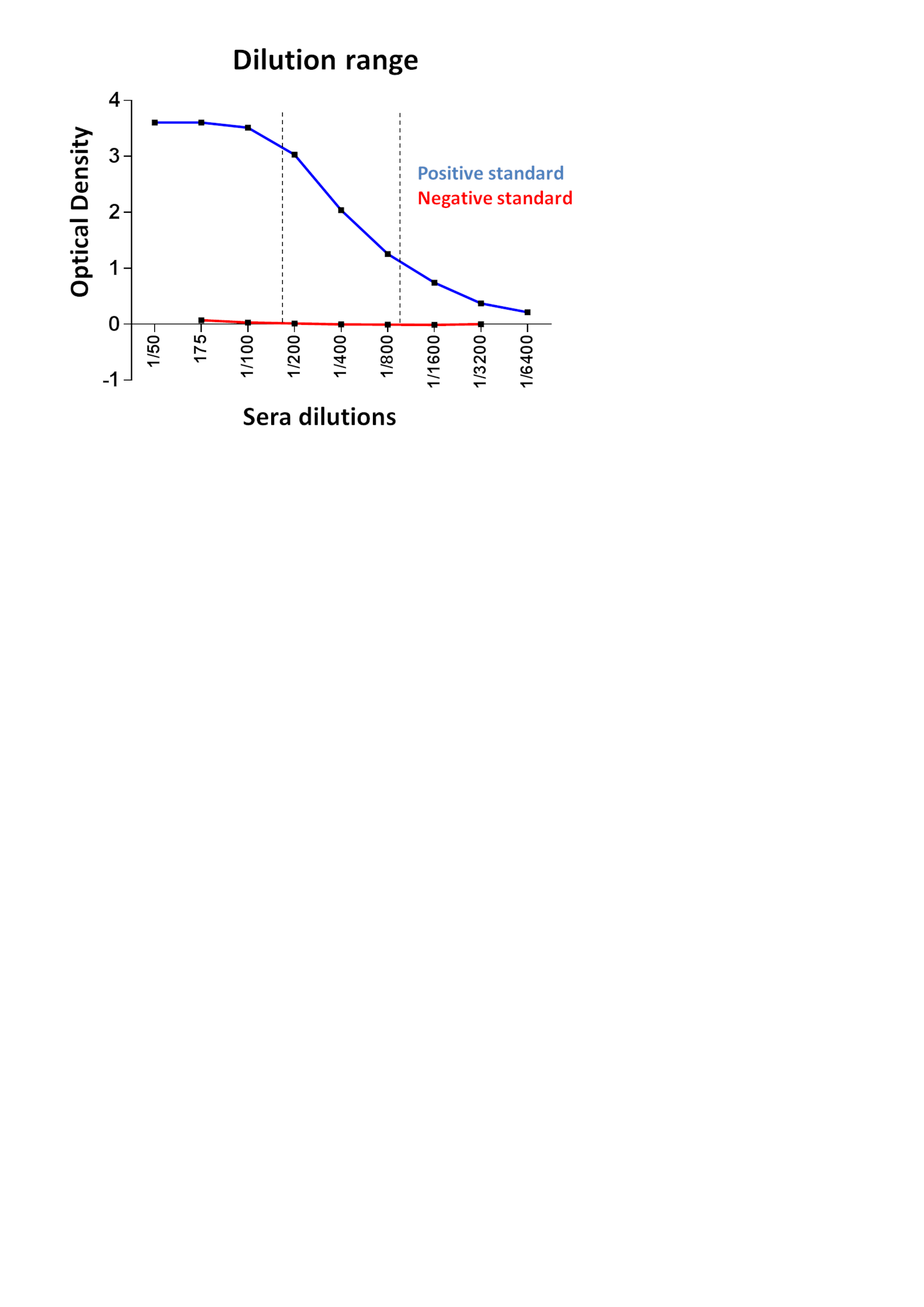

Supplement: Supplementary file 1 — Additional file 1. Serum dilution range of the SOV N ELISA. Serum dilution 1/800 were ultimately selected for antibody detection. [file 13567_2018_615_MOESM1_ESM.tiff]

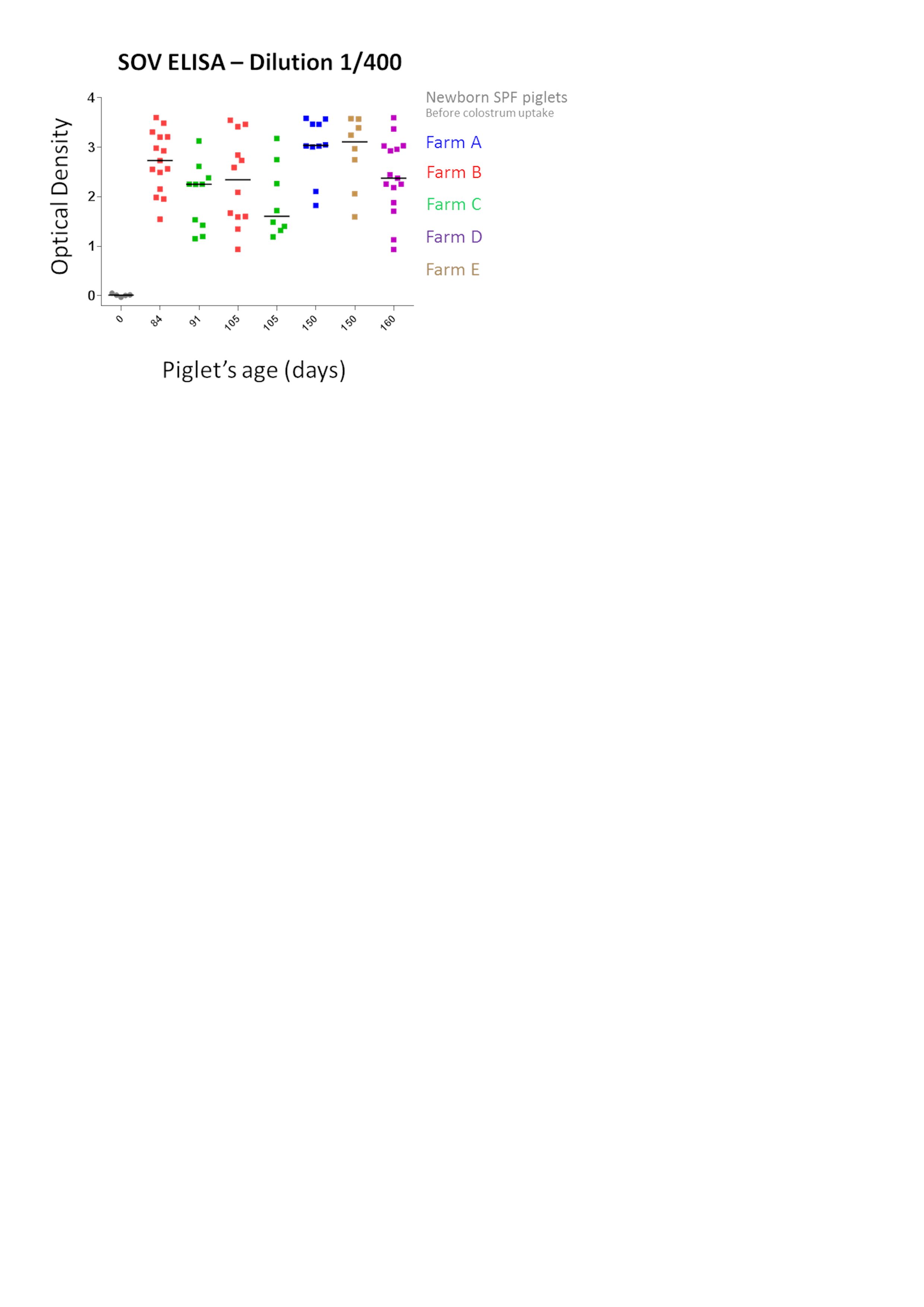

Supplement: Supplementary file 2 — Additional file 2. SOV N ELISA results using dilution 1/400. Optical density (OD) was varying between herds with some specific pathogen free piglets before colostrum uptake staying free of anti-pneumovirus antibodies. [file 13567_2018_615_MOESM2_ESM.tiff]

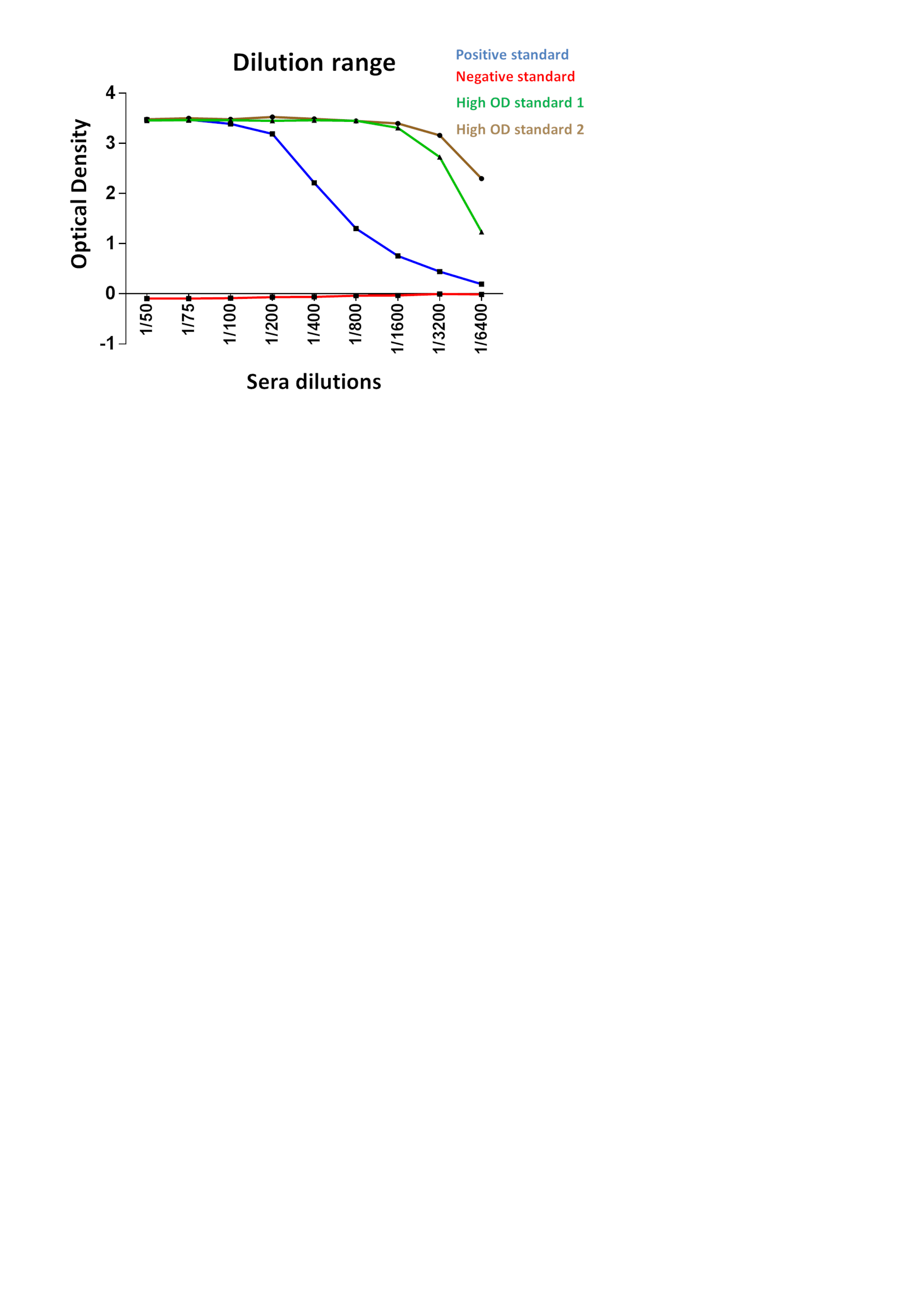

Supplement: Supplementary file 3 — Additional file 3. Standard curves generated from highest OD samples (green and brown lines) were compared to the selected standard curve (blue line). Dilutions of the negative standard is presented in red. [file 13567_2018_615_MOESM3_ESM.tiff]
